# Prevalence of Cannabis Use Disorder and Reasons for Use Among Adults in a US State Where Recreational Cannabis Use Is Legal

**DOI:** 10.1001/jamanetworkopen.2023.28934

**Published:** 2023-08-29

**Authors:** Gwen T. Lapham, Theresa E. Matson, Jennifer F. Bobb, Casey Luce, Malia M. Oliver, Leah K. Hamilton, Katharine A. Bradley

**Affiliations:** 1 Kaiser Permanente Washington Health Research Institute, Seattle; 2 Department of Health Systems and Population Health, University of Washington, Seattle; 3 Department of Medicine, University of Washington, Seattle

## Abstract

**Importance:**

Medical and nonmedical cannabis use and cannabis use disorders (CUD) have increased with increasing cannabis legalization. However, the prevalence of CUD among primary care patients who use cannabis for medical or nonmedical reasons is unknown for patients in states with legal recreational use.

**Objective:**

To estimate the prevalence and severity of CUD among patients who report medical use only, nonmedical use only, and both reasons for cannabis use in a state with legal recreational use.

**Design, Setting, and Participants:**

This cross-sectional survey study took place at an integrated health system in Washington State. Among 108 950 adult patients who completed routine cannabis screening from March 2019 to September 2019, 5000 were selected for a confidential cannabis survey using stratified random sampling for frequency of past-year cannabis use and race and ethnicity. Among 1688 respondents, 1463 reporting past 30-day cannabis use were included in the study.

**Exposure:**

Patient survey–reported reason for cannabis use in the past 30 days: medical use only, nonmedical use only, and both reasons.

**Main Outcomes and Measures:**

Patient responses to the Composite International Diagnostic Interview-Substance Abuse Module for CUD, corresponding to *Diagnostic and Statistical Manual of Mental Disorders, Fifth Edition* CUD severity (0-11 symptoms) were categorized as any CUD (≥2 symptoms) and moderate to severe CUD (≥4 symptoms). Adjusted analyses were weighted for survey stratification and nonresponse for primary care population estimates and compared prevalence of CUD across reasons for cannabis use.

**Results:**

Of 1463 included primary care patients (weighted mean [SD] age, 47.4 [16.8] years; 748 [weighted proportion, 61.9%] female) who used cannabis, 42.4% (95% CI, 31.2%-54.3%) reported medical use only, 25.1% (95% CI, 17.8%-34.2%) nonmedical use only, and 32.5% (95% CI, 25.3%-40.8%) both reasons for use. The prevalence of CUD was 21.3% (95% CI, 15.4%-28.6%) and did not vary across groups. The prevalence of moderate to severe CUD was 6.5% (95% CI, 5.0%-8.6%) and differed across groups: 1.3% (95% CI, 0.0%-2.8%) for medical use, 7.2% (95% CI, 3.9%-10.4%) for nonmedical use, and 7.5% (95% CI, 5.7%-9.4%) for both reasons for use (*P* = .01).

**Conclusions and Relevance:**

In this cross-sectional study of primary care patients in a state with legal recreational cannabis use, CUD was common among patients who used cannabis. Moderate to severe CUD was more prevalent among patients who reported any nonmedical use. These results underscore the importance of assessing patient cannabis use and CUD symptoms in medical settings.

## Key Points

**Question:**

What is the prevalence of cannabis use disorder (CUD) among primary care patients who use cannabis in a state with legal recreational cannabis use, and does prevalence differ by reason for use?

**Findings:**

In this cross-sectional study, weighted prevalence of any CUD did not vary by reason for cannabis use, whereas the prevalence of moderate to severe CUD did. Prevalence of moderate to severe CUD was higher in those who reported nonmedical use only or both medical and nonmedical use.

**Meaning:**

In this study, CUD was common among patients who use cannabis in a state with legal recreational cannabis use, with moderate to severe CUD most prevalent among patients with any nonmedical use.

## Teaser

This cross-sectional study evaluates the prevalence and severity of cannabis use disorder and the reasons reported for use among adults in a US state where recreational cannabis use is legal.

## Introduction

Cannabis use is prevalent and increasing in the US.^1,2,3^ As of June 2023, 38 states have legalized medical cannabis use; among these, 23 have legalized recreational use.^4^ Cannabis use is most prevalent in states with legal recreational use, where now more than half of US adults have legal access to cannabis.^5,6,7,8^ Medical use of cannabis is also growing,^9^ although prevalence varies depending on state law, clinician recommendation, and patient viewpoints.^10,11,12,13^ Among primary care patients who used cannabis in a state with legal recreational use, where provider recommendation is unnecessary for medical use, the prevalence of patient-reported medical cannabis use was 67%.^13^

Greater cannabis use is associated with an increase in risk of cannabis use disorder (CUD),^14,15,16,17^ and legalization has contributed to increases,^18,19,20^ with 17% of individuals who use cannabis having CUD.^21^ Among veteran outpatients who used cannabis in a state with legal medical use, the prevalence of CUD varied by reasons for use and was lowest among those who reported medical use only.^12^ This study assessed whether CUD prevalence varied for patients by self-reported reason for cannabis use in a state with legal recreational use. We estimated the prevalence of CUD based on a diagnostic questionnaire on a confidential cannabis survey among patients who used cannabis, overall and by 3 categories of patient-reported reasons for use: medical use only, nonmedical use only, and both.

## Methods

### Sample

Data for this cross-sectional study were from patients’ survey responses and electronic health records. Demographic characteristics (ie, age, sex, race, ethnicity, and insurance) were collected from patients on paper or via patient portal and documented in the electronic health record by the Kaiser Permanente Washington health system before or at the time of screening. The Kaiser Permanente Washington institutional review board approved the study, including waivers of consent (for sample identification) and documentation of consent (survey respondents). The study followed the Strengthening the Reporting of Observational Studies in Epidemiology (STROBE) reporting guideline.

The study was conducted in Kaiser Permanente Washington, a large health system in Washington State where recreational cannabis use has been legal since 2012. Primary care patients who completed a confidential survey about cannabis use were included in the study. Survey design, sampling, procedures, weighting, and sample characterization, including comparison of respondents to eligible primary care sample and nonrespondents, have been reported previously.^13^ Briefly, 5000 patients 18 years and older were randomly selected from 108 950 eligible patients with electronic health record documentation of completing a cannabis screen as part of routine primary care from March 28, 2019, to September 12, 2019 (Figure). The single-item screen asks about the frequency of past-year cannabis use (ie, none, less than monthly, monthly, weekly, and daily).^22^ Sample selection included patients who reported no past-year use as well as stratified oversampling of patients with more frequent cannabis use and patients of minoritized racial and ethnic groups (including American Indian or Alaska Native, Asian, Black, Hispanic, and Native Hawaiian or Other Pacific Islander) in order to obtain representation of subgroups that are often underrepresented in research.^13^ Race and ethnicity were evaluated as proxies for lived experiences (eg, discrimination) that may influence reasons for or patterns of use. Among invited participants, 1688 (34%) provided informed consent and responded to the survey. Respondents were asked about past-year use and more specific questions about past 30-day use, including reasons, mode, and typical frequency of cannabis use. Patients who reported past 30-day cannabis use (n = 1463) were included here (Figure), with results weighted to the primary care sample who used cannabis in the past 30 days (hereafter, patients who used cannabis).

**Figure.**
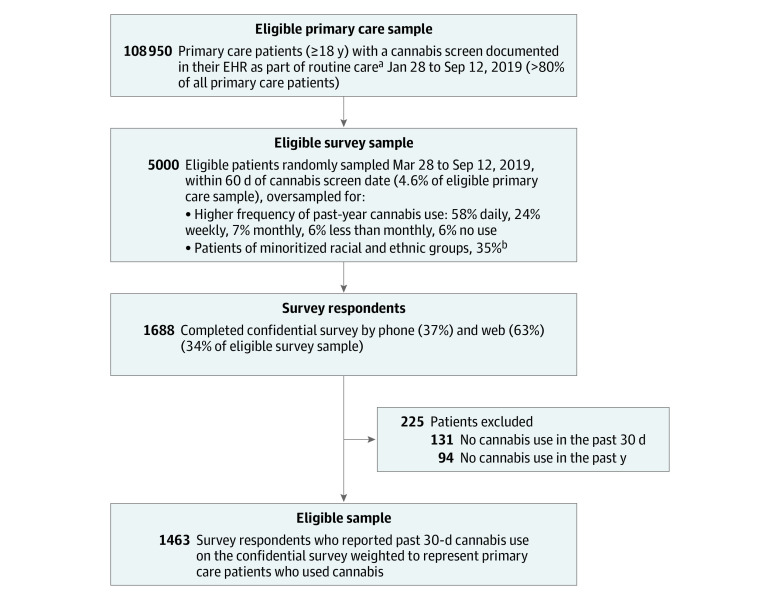
Flow Diagram of Study Sample EHR indicates electronic health record. ^a^Patients who were Kaiser Permanente Washington employees, needed an interpreter, lived outside of Washington state, were recently deceased, or had opted out of EHR were excluded. ^b^Includes American Indian or Alaska Native, Asian, Black, Hispanic, and Native Hawaiian or Other Pacific Islander; oversampling was done to obtain representation of subgroups that are often underrepresented in research.

### Cannabis Survey Measures

Patients were categorized by their stated reason for using cannabis during the past 30 days: medical use only, nonmedical use only, or both reasons for use. All patients who reported past 30-day use were asked the 15-item Composite International Diagnostic Interview Substance Abuse Module (CIDI-SAM). The CIDI-SAM provides a scaled score (0-11 symptoms) of *Diagnostic and Statistical Manual of Mental Disorders, Fifth Edition* (*DSM-5*) CUD severity reflecting the number of past-year *DSM-5* CUD criteria (ie, 2-3 symptoms = mild, 4-5 symptoms = moderate, 6-11 symptoms = severe).^23^ The 11 symptoms are reported, as well as any CUD (≥2 symptoms) and moderate to severe CUD (≥4 symptoms).^22^

Patients were asked about all modes of cannabis use as well as the primary mode, including inhalation (ie, smoke, vape, dab), ingestion (ie, eat, drink), application (ie, lotion, ointment), or other modes. Questions also included the frequency of past-year use and typical number of days per week and times per day of cannabis use.

### Statistical Analysis

All analyses were weighted for sampling strategy and nonresponse to provide estimates reflecting the primary care population who used cannabis.^13^ We report the prevalence of reasons for cannabis use as well as the characteristics of the sample, overall and stratified by reasons for cannabis use. Main analyses estimated the prevalence of cannabis measures and CUD symptoms and severity across reported reasons for cannabis use, using multinomial logistic regression for categorical outcomes and logistic regression for binary outcomes, with robust standard errors (SEs) and adjustment for age, sex, race, ethnicity, and insurance status. Results are presented as the mean predicted probability of outcomes based on recycled predictions; corresponding SEs were estimated using the delta method, which assumes a normal approximation to construct 95% CIs.^24,25^ Joint Wald tests evaluated differences in CUD across reasons for use. Post hoc sensitivity analyses repeated analyses in a restricted sample excluding patients who reported applied use (topical) as their only mode of cannabis use, due to the expected lower risk of CUD from limited systemic cannabis exposure.^26^ Analyses were conducted between August and June 2023 using Stata version 17.0 (StataCorp). Two-sided *P* < .05 was considered statistically significant.

## Results

The primary care sample of patients who used cannabis (n = 1463 patient respondents weighted to primary care population who used cannabis) was predominantly middle-aged (weighted mean [SD] age, 47.4 [16.8] years); female (748 [weighted proportion, 61.9%] vs 715 male [weighted proportion, 38.1%]), and commercially insured (935 [61.1%]) (Table 1). By self-report, 12 patients (weighted proportion, 0.2%) were American Indian or Alaska Native, 56 (weighted proportion, 2.4%) were Asian, 120 (weighted proportion, 4.9%) were Black, 10 (weighted proportion, 0.2%) were Native Hawaiian or Other Pacific Islander, 1029 (weighted proportion, 78.6%) were White, 95 (weighted proportion, 5.3%) reported multiple races, and 141 (weighted proportion, 8.3%) were of another or unknown race; 151 (weighted proportion, 3.5%) reported Hispanic ethnicity and 1239 (weighted proportion, 9.3%) non-Hispanic ethnicity.

**Table 1.** Characteristics of Primary Care Patients Who Used Cannabis

	% (SE)^a^				*P* value^c^
	Medical use only (n = 234)^b^	Nonmedical use only (n = 425)^b^	Both reasons for use (n = 804)^b^	Total (N = 1463)^b^	
Sex					
Female	76.5 (8.3)	43.4 (9.4)	57.3 (4.1)	61.9 (4.8)	.02
Male	23.5 (8.3)	56.6 (9.4)	42.7 (4.1)	38.1 (4.8)	
Age, mean (SD), y	53.6 (14.6)	39.9 (16.6)	45.1 (16.8)	47.4 (16.8)	
18-29	3.2 (1.4)	42.4 (9.5)	23.2 (2.9)	19.5 (3.7)	.003
30-44	24.5 (10.3)	24.1 (5.0)	30.9 (3.6)	26.5 (4.7)	
45-64	43.3 (11.3)	17.5 (3.9)	26.0 (3.4)	31.2 (5.3)	
≥65	29.0 (10.1)	16.0 (8.3)	19.9 (5.6)	22.8 (5.1)	
Race^d^					
American Indian or Alaska Native	0.0 (0.0)	0.2 (0.1)	0.5 (0.3)	0.2 (0.1)	.55
Asian	0.4 (0.2)	6.1 (2.2)	2.2 (1.0)	2.4 (0.7)	
Black	5.2 (4.7)	3.2 (0.9)	5.7 (1.4)	4.9 (2.0)	
Multiracial^e^	7.2 (6.6)	2.7 (0.8)	4.9 (1.3)	5.3 (2.8)	
Native Hawaiian or Other Pacific Islander	0.2 (0.1)	0.5 (0.3)	0.2 (0.1)	0.2 (0.1)	
White	78.4 (9.4)	79.0 (4.6)	78.7 (2.7)	78.6 (4.2)	
Other or unknown^e^	8.6 (6.2)	8.3 (2.7)	7.9 (1.3)	8.3 (2.8)	
Ethnicity					
Hispanic	2.0 (1.1)	3.3 (0.8)	5.6 (1.1)	3.5 (0.7)	.09
Non-Hispanic	97.0 (1.5)	89.1 (3.0)	90.2 (1.5)	92.8 (1.1)	
Unknown	0.9 (0.4)	7.6 (2.7)	4.3 (0.9)	3.7 (0.8)	
Insurance					
Medicaid/subsidized	12.1 (6.9)	6.2 (2.0)	10.6 (1.9)	10.1 (3.1)	.29
Medicare	33.7 (10.5)	14.2 (8.3)	20.4 (5.6)	24.5 (5.2)	
Commercial	52.1 (11.3)	73.9 (8.3)	63.0 (5.0)	61.1 (5.4)	
Unknown	2.0 (1.2)	5.7 (2.6)	6.0 (1.8)	4.3 (1.0)	
Education^f^^,^^g^					
High school, GED, or less	19.3 (9.6)	11.3 (2.9)	19.1 (3.0)	17.3 (4.3)	.32
Some college	55.5 (11.2)	35.2 (10.0)	44.6 (4.7)	46.9 (5.6)	
4-y College degree	9.4 (5.5)	26.9 (5.7)	19.7 (2.6)	17.2 (2.9)	
>4-y College degree	15.5 (7.8)	26.5 (8.2)	16.2 (2.7)	18.5 (4.1)	
Employment^f^^,^^g^					
Full time	50.6 (11.3)	53.2 (9.4)	64.4 (3.8)	55.7 (5.5)	.07
Part time	3.7 (1.7)	19.8 (11.4)	6.6 (1.5)	8.7 (3.4)	
Retired	33.6 (10.6)	15.4 (8.3)	14.7 (2.4)	22.9 (5.1)	
Other	11.1 (6.8)	7.6 (2.4)	9.8 (2.1)	9.8 (3.0)	
Unemployed	0.8 (0.4)	3.9 (2.2)	4.1 (1.5)	2.7 (0.8)	
Marital Status^f^^,^^g^					
Married or living with partner	49.7 (11.3)	61.1 (7.4)	61.9 (3.9)	56.5 (5.5)	.45
Divorced or separated	18.4 (9.4)	7.7 (2.9)	9.2 (1.8)	12.7 (4.2)	
Widowed	1.0 (0.7)	0.8 (0.4)	2.7 (1.1)	1.5 (0.5)	
Single or never married	30.4 (10.9)	30.4 (6.1)	25.9 (3.2)	28.9 (5.0)	
Residence^f^^,^^g^					
Own	61.7 (11.1)	62.3 (7.1)	56.6 (4.2)	60.2 (5.2)	.35
Rent	36.6 (11.1)	29.0 (5.7)	38.8 (3.9)	35.4 (5.2)	
No permanent residence/living with friends or family	1.5 (1.0)	8.7 (2.7)	4.3 (1.1)	4.2 (0.9)	

Abbreviations: GED, General Educational Development Test; SE, standard error.

^a^ Percentage and standard errors calculated from unadjusted survey data weighted for sampling and nonresponse rates for eligible primary care sample.

^b^ Sample numbers from unweighted survey data.

^c^ Pearson χ^2^ test of independence.

^d^ Race and ethnicity data were collected via self-report on paper or via an online patient portal and documented by the health system. These variables were included as proxies for lived experiences (eg, discrimination) that may influence reasons for or patterns of use.

^e^ Patients are provided the option to indicate other, which is undefined, when choosing among 1 or more race categories at appointing or check-in. Patients who indicated more than 1 race are reported as multiracial.

^f^ Indicates data from survey; all other data are from the electronic health record.

^g^ Survey responses for education, employment, marital status, and residence were missing for 9, 5, 6, and 7 patients, respectively.

Among patients who used cannabis, the prevalence of patient reasons for cannabis use included 42.4% (95% CI, 31.2%-54.3%) reporting medical use only, 25.1% (95% CI, 17.8%-34.2%) reporting nonmedical use only, and 32.5% (95% CI, 25.3%-40.8%) reporting both reasons for use. Patients reporting medical use only tended to be older (mean [SD] age, 53.6 [14.6] years), were mostly female (142 [76.5%]), retired (67 [33.6%]), and mostly had Medicare (84 [33.7%]). The prevalence of patients who reported any medical cannabis use (ie, medical use only or both reasons for use) was 74.7% (95% CI, 65.7%-82.1%), while the prevalence of any reported nonmedical cannabis use (ie, nonmedical use only or both reasons for use) was 57.5% (95% CI, 45.6%-68.6%).

The prevalence of any CUD was 21.3% (95% CI, 15.4%-28.6%) and did not differ depending on patient reasons for use (Table 2). The prevalence of moderate to severe CUD was 6.5% (95% CI, 5.0%-8.6%) and differed across groups: 1.3% (95% CI, 0.0%-2.8%) for medical use only; 7.2% (95% CI, 3.9%-10.4%) for nonmedical use only; and 7.5% (95% CI, 5.7%-9.4%) both reasons for use (*P* = .01). For all groups, the most prevalent CUD symptoms were tolerance, uncontrolled escalation of use and craving. Compared with patients with medical use only, patients with nonmedical use only or both reasons for use were more likely to report withdrawal, use in hazardous situations, continue use despite consequences, time spent on use, interference with obligations, and activities given up.

**Table 2.** Prevalence of Cannabis Use Disorder by Reason for Cannabis Use Among Primary Care Patients Who Used Cannabis^a^

	Participants, % (95% CI)			*P* value^c^
	Medical use only^b^	Nonmedical use only^b^	Both reasons for use^b^	
Past year *DSM 5* CUD^d^^,^^e^				
Any CUD (≥2 symptoms)	13.4 (0.1-26.7)	22.4 (14.9-30.0)	25.6 (21.0-30.2)	.29
Moderate-severe CUD (≥4 symptoms)	1.3 (0.0-2.8)^f^	7.2 (3.9-10.4)	7.5 (5.7-9.4)	.01
Past year *DSM 5* CUD symptoms^e^				
Tolerance	18.4 (6.0-30.8)	13.3 (7.0-19.6)	19.1 (14.4-23.9)	.37
Uncontrolled escalation of use	13.5 (0.7-26.3)	24.2 (15.4-33.0)	29.1 (23.0-35.2)	.14
Craving	12.5 (0.0-25.0)^f^	18.7 (12.6-24.9)	19.8 (16.1-23.5)	.60
Withdrawal	1.8 (0.2-3.3)	9 (5.2-12.8)	13.5 (10.1-16.9)	<.001
Hazardous situations	1.4 (0.0-2.8)^f^	6.8 (4.0-9.7)	10.4 (8.0-12.8)	<.001
Failed attempts to cut down	0.9 (0.0-2.1)^f^	4.2 (2.5-5.9)	3.2 (2.1-4.3)	.11
Continued use despite consequences	0.4 (0.0-0.9)^f^	4.1 (2.3-5.9)	3.8 (2.4-5.3)	.001
Time spent	0.2 (0.0-0.6)^f^	2.8 (0.7-4.9)	1.5 (0.8-2.1)	.01
Interference with role obligations	0.6 (0.0-1.7)^f^	3.8 (1.9-5.7)	1.7 (0.9-2.5)	.02
Interpersonal problems	0.6 (0.0-1.3)	1.1 (0.4-1.9)	1 (0.4-1.6)	.63
Gave up activities	0.2 (0.0-0.5)^f^	2.8 (1.3-4.3)	2.7 (1.5-3.9)	.007
Any CUD symptom^d^	25.1 (11.1-39.1)	36.2 (25.5-46.9)	50.6 (44.2-57.0)	.003

Abbreviations: CUD, cannabis use disorder; *DSM 5*, *Diagnostic and Statistical Manual of Mental Disorders, Fifth Edition*.

^a^ Data collected from confidential survey, N = 1463.

^b^ *P* value based on a joint Wald test from logistic regression.

^c^ Mean predicted probabilities were estimated from logistic regression models fit to the survey data and weighted for sampling and nonresponse rates (adjusted for age, sex, race and ethnicity, and insurance status).

^d^ Any past-year CUD-based *DSM-5* criteria.

^e^ The 15-item Composite International Diagnostic Interview Substance Abuse Module (CIDI-SAM) for *DSM 5* CUD was offered to all respondents who reported past 30-day cannabis use and provides a scaled score of *DSM 5* CUD severity (0-11 symptoms) reflecting the number of *DSM 5* CUD criteria met.

^f^ Estimates of confidence intervals were not bound at 0; negative values were truncated at zero.

The most common primary mode of use was application for patients with medical use only, 36.1% (95% CI, 21.2%-51.0%), and inhalation for patients with nonmedical use only and both reasons for use, 59.5% (95% CI, 43.5%-75.6%) and 69.4% (95% CI, 60.4%-78.5%), respectively (Table 3). Patients who reported both reasons for use were more likely to use cannabis 3 or more times per day (95% CI, 39.7% vs 4.5%-14.0%), with 71.8% using cannabis 4 or more days per week (95% CI, 63.7%-79.9%). Post hoc sensitivity analyses to remove patients whose only mode of cannabis use was applied (n = 22) did not meaningfully impact the prevalence of any CUD or moderate to severe CUD, 25.0% (95% CI, 18.3%-33.0%) and 7.7% (95% CI, 5.9%-10.0%), respectively, or differences in prevalence by reason for use, and did not change results of significance testing (eTable 1 in Supplement 1).

**Table 3.** Prevalence of Cannabis Use Characteristics by Reason for Cannabis Use Among Primary Care Patients Who Used Cannabis

	Participants, % (95% CI)		
	Medical use only^a^	Nonmedical use only^a^	Both reasons for use^a^
Mode of use^b^			
Smoke	18.9 (2.2-35.5)	46.2 (31.2-61.3)	74.1 (64.6-83.7)
Vape	8.7 (3.3-14.2)	38.1 (21.5-54.6)	41.5 (33.7-49.2)
Dab	3.3 (1-5.5)	4.3 (2-6.7)	9.5 (7.3-11.6)
Eat	33.8 (14.5-53.1)	61.9 (45.8-78)	41.9 (34.4-49.5)
Drink	7.2 (1.9-12.6)	6.8 (2.6-11)	16.1 (10.5-21.8)
Apply	61.3 (43-79.5)	13 (5.6-20.3)	46.3 (39.1-54.4
Other	11 (1-20.9)	5.1 (0.0-14.2)^c^	2.1 (0.4-3.8)
Modes of use, mean (SD)	1.5 (1.3-1.7)	1.8 (1.5-2.1)	2.3 (2.2-2.4)
Primary mode of use^b^			
Inhalation (smoke, vape, dab)	25.5 (9-42.1)	59.5 (43.5-75.6)	69.4 (60.4-78.5)
Ingestion (eat, drink)	26.8 (10.3-43.3)	37.5 (21.8-53.3)	18.3 (11.3-25.3)
Application (lotions, ointments)	36.1 (21.2-51)	0.6 (0.0-1.5)^c^	11.1 (4.9-17.3)
Other (sublingual, lozenge)	11.4 (1.5-21.3)	2.3 (−3.2-7.9)	0.6 (0.0-1.4)^c^
Typical d/wk of use^b^^,^^d^			
<1	17.7 (1.7-33.7)	27.2 (14.2-40.2)	8 (3.1-12.8)
1-3	24.9 (6.8-42.9)	39.3 (23.5-55.1)	20.2 (13.3-27)
4-7	57.4 (41-73.8)	33.5 (20.5-46.5)	71.8 (63.7-79.9)
Typical times/d of use^b^^,^^d^			
<1	12.1 (0.0-26.0)^c^	12.3 (2.4-22.2)	2 (0.5-3.4)
1-2	83.4 (69.3-97.5)	73.7 (63.7-83.7)	58.3 (47.6-69.0)
≥3	4.5 (2.0-7.0)	14 (8.7-19.4)	39.7 (28.8-50.6)
Frequency of past year use			
<Monthly	12.5 (2.8-22.2)	20.1 (3.3-36.9)	5.4 (0.8-10)
Monthly	32.8 (19.4-46.3)	18.2 (9.3-27.1)	10.3 (5.3-15.3)
Weekly	15.8 (9-22.6)	36.6 (23.7-49.4)	28.2 (21.2-35.2)
Daily or almost daily	38.8 (23.9-53.8)	25.2 (14.1-36.3)	56.1 (48.3-64)

^a^ Percentages and 95% CIs calculated from unadjusted survey data weighted for sampling and nonresponse rates for eligible primary care sample.

^b^ Pearson χ^2^ test of independence.

^c^ Estimates of confidence intervals were not bound at 0; negative values were truncated at zero.

^d^ Patients are provided the option to indicate other, which is undefined, when choosing among 1 or more race categories at appointing or check-in. Patients who indicated more than 1 race are reported as multiracial.

## Discussion

In this cross-sectional study of primary care patients in a state with legal recreational cannabis use, CUD was common among patients who used cannabis, with 21% having CUD and 6% having moderate to severe CUD. Patients who used cannabis for medical reasons only were mostly older and likely to use applied products. Patients who reported any nonmedical use were at greatest risk of moderate to severe CUD (7.2% to 7.5%). While the prevalence of moderate to severe CUD was lowest among patients who reported medical use only (1.3%), 13.4% met criteria for mild, moderate, or severe CUD.

The prevalence of CUD among patients who use cannabis found here is comparable to recent studies of patients who use cannabis in states with legal medical and recreational use.^12,27^ Moreover, comparable to the study by Browne et al,^12^ the prevalence of moderate to severe CUD in this study was significantly lower for patients reporting medical use only.

The finding that CUD was common among primary care patients in a state with legal recreational use, where more than 20% of the population reports cannabis use,^13^ underscores the importance of assessing patient cannabis use in clinical settings. Population-based screening with a validated single-item screen can identify patients who use cannabis and may be at risk of CUD.^22^ Knowledge of patient use provides an opportunity to discuss risks and limited benefits of cannabis use and potentially safer treatment alternatives for those using cannabis for medical reasons.^28^ For patients with higher risk cannabis use (eg, daily), psychometrically valid brief assessments for *DSM-5* symptoms of CUD can identify and gauge CUD severity.^29^ Such knowledge can support engagement around symptoms, shared decision-making, and offering of treatment if desired, especially for patients with moderate to severe CUD who may benefit most from treatment. Yet research is needed on how best to assess and document patient reasons for cannabis use and to engage individuals with CUD in treatment.

### Limitations

Study limitations include a lower than desired survey response rate (34%), consistent with declining national averages.^30,31^ Small cell sizes of respondents with moderate to severe CUD could have influenced results; however, oversampling of patients reporting higher frequency cannabis use reduces this concern (eTable 2 in Supplement 1). Respondents reflect the demographic characteristics of primary care patients in 1 health system, and results may not generalize to other patients, including those in states with different cannabis laws.^13,22^ While the survey sample supported current analyses, small subgroups restricted exploration of CUD prevalence by patient reasons for use across important patient demographic characteristics (eg, age, sex, race, ethnicity) and cannabis measures (eg, mode and frequency of use). Thus further exploration is needed, along with longitudinal studies, to understand associations between patient characteristics, reasons for use, and CUD risk at the point-of-care and over time.

## Conclusions

In this study, CUD was common (21%) among primary care patients who use cannabis in a state with legal recreational use, with patients using for nonmedical reasons most at risk of moderate to severe CUD. As legal recreational cannabis use among adults continues to increase across the US, the results here underscore the importance of assessing patient cannabis use and CUD symptoms in medical settings.

## Notes

Supplement 1. **eTable 1.** Sensitivity analysis: Prevalence of cannabis use disorder by reason for cannabis use among primary care patients who used cannabis, with removal of patients (n=22) who reported applied use as their only mode of use **eTable 2.** Unweighted crosstabulation of survey participants with past 30-day cannabis use who reported cannabis use disorder symptoms, by reason for cannabis use

Supplement 2.
Data Sharing Statement
