# Supplementary material for: Prevalence of Cannabis Use Disorder and Reasons for Use Among Adults in a US State Where Recreational Cannabis Use Is Legal
Source: JAMA Netw Open. 2023 Aug 29;6(8):e2328934. doi: 10.1001/jamanetworkopen.2023.28934 (PMC10466162; doi:10.1001/jamanetworkopen.2023.28934)
Supplement: Supplement 1. — eTable 1. Sensitivity analysis: Prevalence of cannabis use disorder by reason for cannabis use among primary care patients who used cannabis, with removal of patients (n=22) who reported applied use as their only mode of use eTable 2. Unweighted crosstabulation of survey participants with past 30-day cannabis use who reported cannabis use disorder symptoms, by reason for cannabis use [file jamanetwopen-e2328934-s001.pdf]

Supplemental Online Content

Lapham GT, Matson TE, Bobb JF, et al. Prevalence of cannabis use disorder and reasons for use among adults in a us state where recreational cannabis use is legal. *JAMA Netw Open*. 2023;6(8):e2328934. doi:10.1001/jamanetworkopen.2023.28934

eTable 1. Sensitivity analysis: Prevalence of cannabis use disorder by reason for cannabis use among primary care patients who used cannabis, with removal of patients (n=22) who reported applied use as their only mode of use

eTable 2. Unweighted crosstabulation of survey participants with past 30-day cannabis use who reported cannabis use disorder symptoms, by reason for cannabis use

This supplemental material has been provided by the authors to give readers additional information about their work.

© 2023 Lapham GT et al. *JAMA Network Open.*

eTable 1. Sensitivity analysis: Prevalence of cannabis use disorder by reason for cannabis use among primary care patients who used cannabis, with removal of patients (n=22) who reported applied use as their only mode of use[a]

	Medical use only[b]		Nonmedical use only[b]		Both reasons for use[b]		
	%	*(95% CI)*	*%*	*(95% CI)*	*%*	*(95% CI)*	*p-value*[c]
**Past-year DSM 5 CUD**[d]							
Any CUD (≥2 symptoms)[e]	17.5	(1.5-33.6)	24.4	(16.4-32.5)	27.5	(22.7-32.3)	0.505
Moderate-severe CUD (≥4 symptoms)[e]	1.7	(0.0*-3.7)	8.0	(4.4-11.5)	8.3	(6.3- 10.3)	**0.026**

[a]Average predicted probabilities were estimates from multinomial logistic regression models fit to and weighted for sampling and nonresponse rates (adjusted for age, sex, race and ethnicity, and insurance status); [b]Number of patients whose primary mode of use was applied products (n=22) and removed from analyses were 21, 0, and 1, for patients with medical use only, nonmedical use only, and both reasons for use, respectively (total n-1,441); [c] p-value based on a joint Wald test from logistic regression, bolding indicates statistical significance for p-values <0.05; [d]15-item Composite International Diagnostic Interview Substance Abuse Module (CIDI-SAM) for DSM 5 CUD was offered to all respondents who reported past 30-day cannabis use and provides a scaled score of DSM 5 CUD severity (0-11 symptoms) reflecting the number of DSM 5 CUD criteria met; [e] Indication of any past-year CUD based DSM 5 criteria; CUD=cannabis use disorder; DSM 5=Diagnostic and Statistical Manual of Mental Disorders, Fifth Edition;

© 2023 Lapham GT et al. *JAMA Network Open.*

eTable 2. Unweighted crosstabulation of survey participants with past 30-day cannabis use who reported cannabis use disorder symptoms, by reason for cannabis use

	Medical use only	Nonmedical use only	Both reasons for use	Total
	(n=234)	(n=425)	(n=804)	(n=1,463)
	N	N	N	N
**Past-year DSM 5 CUD**[a]				
No CUD	195	120	349	664
Any CUD (≥2 symptoms)[b]	31	205	334	570
Moderate-severe CUD (≥4 symptoms)[b]	8	100	121	229

[b]15-item Composite International Diagnostic Interview Substance Abuse Module (CIDI-SAM) for DSM 5 CUD was offered to all respondents who reported past 30-day cannabis use and provides a scaled score of DSM 5 CUD severity (0-11 symptoms) reflecting the number of DSM 5 CUD criteria met; [b] Indication of any past-year CUD based DSM 5 criteria; CUD=cannabis use disorder; DSM 5=Diagnostic and Statistical Manual of Mental Disorders, Fifth Edition;

© 2023 Lapham GT et al. *JAMA Network Open.*
