# Supplementary material for: Prevalence of Cannabis Use Disorder and Reasons for Use Among Adults in a US State Where Recreational Cannabis Use Is Legal
Source: JAMA Netw Open. 2023 Aug 29;6(8):e2328934. doi: 10.1001/jamanetworkopen.2023.28934 (PMC10466162; doi:10.1001/jamanetworkopen.2023.28934)
Supplement: Supplement 2. — Data Sharing Statement [file jamanetwopen-e2328934-s002.pdf]

Data Sharing Statement

Lapham. Prevalence of Cannabis Use Disorder and Reasons for Use Among Adults in a US State Where Recreational Cannabis Use Is Legal. *JAMA Netw Open*. Published August 29, 2023. doi:10.1001/jamanetworkopen.2023.28934

Data
Data available: No
